# Postpartum Care Behavior Improvement during COVID-19 Pandemic in Indonesia Using Mobile-Health Interactive Message

**DOI:** 10.4314/ejhs.v32i2.4

**Published:** 2022-03

**Authors:** Respati Wulandari, Agus Suwandono, Martha Irene Kartasurya, Sri Achadi Nugraheni

**Affiliations:** 1 Doctoral Program, Faculty of Public Health, Diponegoro University, Semarang, Indonesia; 2 Departement of Epidemiology, Faculty of Public Health, Diponegoro University, Semarang, Indonesia; 3 Department of Public Health Nutrition, Faculty of Public Health, Diponegoro University, Semarang, Indonesia

**Keywords:** Text messaging, Health Promotion, COVID-19, Postpartum Care

## Abstract

**Background:**

Since 2018, maternal mortality in Semarang City, Indonesia, has mostly (75%) occurred during the postpartum period. Therefore, a health intervention is necessary to improve safe and effective postpartum care. During the Covid-19 pandemic, a mobile-based health intervention is preferred due to the government's regulation of COVID-19 safety prevention. This study aimed to determine the effectiveness of the mobile-health interactive message on the postpartum care behavior of mothers and their husbands.

**Methods:**

The study was conducted in a quasi-experimental design. It includes the treatment group and the control group, in which each group consists of 46 pairs of pregnant women in the third trimester and their husbands. The research subjects were chosen through the purposive sampling technique. Data collection was conducted via interviews and observations. The m-health intervention is carried out in the form of text messages, images, videos, and interactive mentoring. The latter was carried out through group messaging via the WhatsApp application for the treatment group. Meanwhile, the control group received regular counseling from the local Community Health Center. Data analysis was performed by Mann-Whitney test, unpaired T-Test, Chi-Square Test dan Fisher Exact Test.

**Results:**

Intervention for 2.5 months increased the knowledge of mothers and husbands. The intervention for 3.5 months improved the mother's attitude, but not the husband's attitude. The intervention also improves maternal practices related to postpartum visits, such as Early Initiation of Breastfeeding assistance requests from health workers, iron tablets and nutritious food consumption, personal hygiene, postpartum danger signs monitoring, and the husband's practice of accompanying mothers during postpartum visits.

**Conclusion:**

Mobile-Health interactive messages effectively improved postpartum care behavior for mothers and their husbands.

## Introduction

The postpartum period is a risky period where maternal and infant mortality globally occurs in the first month, namely in the first week after giving birth (1). Seventy-five percents of maternal and infant deaths occur at the beginning of postpartum in developing countries, including Indonesia.

Central Java Province is one of the provinces which has a highly significant maternal mortality rate in Indonesia. In 2019, Central Java Province contributed to 25.72% of postpartum maternal deaths at the national level. The highest causes of maternal mortality were hypertension in pregnancy (29.6%) and bleeding (24.5%) (2) (3). Semarang City is the capital city of Central Java Province which ranks the 4th largest for maternal mortality (3). In 2015–2018, maternal mortality during the postpartum period was quite significant, namely 75% (4).

Most maternal mortality is preventable (5). Maternal health care is a solution to prevent and overcome the problem of postpartum complications (6). Standard postpartum care checks in Indonesia include routine health checks (examination of vital signs, lochia, bleeding, signs of infection, breasts, uterine contractions, uterine fundus), communication, information and education regarding exclusive breastfeeding and the use of contraceptives, personal hygiene, nutrition, vitamins A, iron tablets, and puerperal danger signs. Increased knowledge about postpartum care is important for mothers to successfully pass the critical period of postpartum (7). Improving postpartum care behavior can be done through innovative information, education, and counseling programs that target mothers and families (8).

The findings of several previous studies in Indonesia stated that mothers' knowledge in postpartum care was low (8). There was a gap in knowledge of postpartum care, especially for mothers who lived in rural areas and had low education (9). The Indonesian government's used maternal and child health book as an education and communication media for mothers, but it did not help in increasing maternal knowledge (10). Maternal and Child Health Surveillance special officers are trying to suppress the maternal mortality rate in Semarang City with personal maternal assistance. However, this effort has not met sufficient expectations because their work performance is not optimal (11).

Corona virus-19 pandemic has changed the way of health promotion implementation, especially through the health protocols' requirements. The use of information technology-based health promotion is one of the solutions (12). This study utilizes m-Health to improve the postpartum care behavior of mothers and their partners in Semarang City, Indonesia.

## Materials and Methods

**Design and research subject**: This research was conducted with a quasi-experimental design in two working areas of Bandarharjo Community Health Center and Bangetayu Community Health Center, Semarang City, Indonesia. The research was conducted from August 2020 to January 2021. The subjects were selected purposively from data on pregnancy visits at the Community Health Center. Inclusion criteria for mothers: gestational age of 28–34 weeks, living with their husband, not having a plan to give birth outside of the city, having a mobile phone with WhatsApp application, willing to participate in this study. The exclusion criteria are the mothers who had a stillbirth, the mother who died due to childbirth, and the mother or husband who moved/ gave birth outside Semarang city.

**Intervention**: Interactive m-health message intervention was used in the form of flyers (text, images), videos, and assistance (consultation, discussion, sharing, and question and answer) using WhatsApp groups. Studies in the preparation of intervention materials have been published previously (13). The intervention was carried out every day, 5 hours/day, for 14 days, followed by random flyers from delivery to 42 days after deliveries. Thus, the overall intervention period was 14 weeks. The treatment group was divided into 5 WhatsApp groups, each group consisting of 9–10 pairs of mothers and their partners and a Maternal and Child Health Surveillance special officers. She is the facilitator of the group and the companion for consultation during the intervention. The controls were not included in the WhatsApp group and received regular counseling from the local Community Health Center.

**Data collection and follow-up**: Data collection was conducted by interviews and observations. Instruments in the form of questionnaires and observation sheets were tested for validity and reliability. Data collection was divided into three stages applicable to the treatment and control groups, namely: before intervention (pre-test I), 2.5 months of intervention (post-test I), and 3.5 months of intervention (post-test II). Before the intervention, the knowledge, attitudes scores, and the characteristics of mothers and husbands were collected (pre-test). After 2.5 months of intervention, measurements were made on the mothers for knowledge, attitudes, and practices, while for the husbands, measurements were made for knowledge, attitudes, and support (post-test 1). Finally, after 3.5 months of intervention, the mothers were again measured for knowledge, attitude, and practices, while the husbands were also re-measured for knowledge, attitude, support, and practice (post-test 2).

**Sample size**: In obtaining two scores of difference in the knowledge of postpartum care, with a power of 80% and a significant level of 5%, the minimum sample size is 33 pairs for each group (14). This study added 40% to the minimal sample size to anticipate dropout, so the samples for each group were 46 pairs of mothers and husbands.

**Variables**: The dependent variables from the mothers' subjects include knowledge, attitudes, and practices of: 1) requesting Early Initiation of Breastfeeding to health workers, 2) monitoring the danger signs of postpartum, 3) consuming vitamin A capsules, 4) conducting postpartum visits at least four times, 5) giving exclusive breastfeeding at least for 42 days, 6) consuming nutritious food and an adequate drinks, 7) consuming iron tablets, 8) using postnatal contraception, 9) maintaining personal hygiene and daily care. The dependent variables from the husbands' subjects were the knowledge, attitude, support, and practices of accompanying their wives in postpartum visits. The confounding variables considered in this study include maternal characteristics (age, education, occupation, income, health insurance ownership, parity, and information exposure) and husbands' characteristics (age, education, occupation, income, and information exposure). After comparison analysis between the groups, the confounding variables found in this study were education and occupation.

**Data processing and analysis**: Data were checked for completeness and analyzed using the Statistical Package for Social Science version 23.0. The Chi-Square test and Fisher's Exact test were used to determine the differences in the characteristics of the subjects in the two groups. The Mann-Whitney test and unpaired t-test were used to determine differences in numerical data on postpartum care behavior variables. Meanwhile, the categorical variables were analyzed using the Chi-Square test and Fisher's Exact test. The strength of the association of intervention and postpartum care behavior was interpreted using the relative risk at 95% CI. The significance criteria at p-value < 0.05.

**Research ethics**: All subjects in this study had received informed consent. Ethical clearance has been obtained from the Ethical Commission of Public Health Faculty, Diponegoro University with the number: 237/EA/KEPK-FKM/2020.

## Results

**Figure 1:**
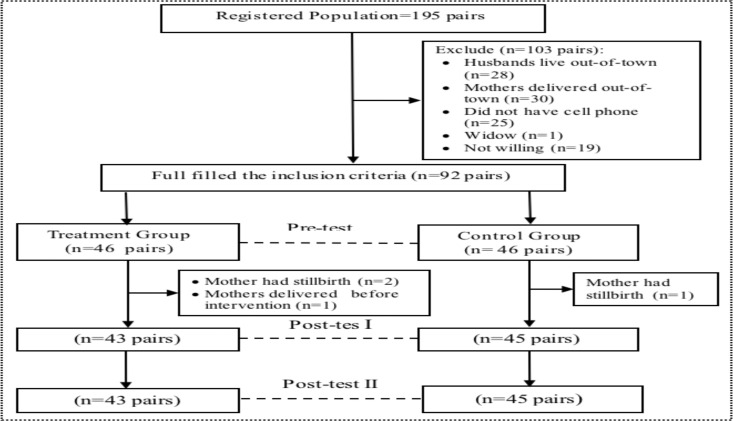
Consort table

**Socio-demographic characteristics**: A total of 43 pairs (mothers and their husbands) in the treatment group and 45 pairs in the control group finished this study (Figure 1).

Table 1 shows the characteristics of mothers and their husbands in both groups. The majority of mothers and husbands were between the age of 20–35 years. Most of the mothers were non-working, having 1–2 children and health insurance. Information exposure about postpartum care were mainly obtained from digital information. There was no difference in the characteristics of the mother and husband in the treatment group and the control group. Therefore, the considered confounding variables did not exist in this study.

**Table 1:** Subjects' characteristics

Variable	Mother n=43			Husband n=45		

	Intervention group f (%)	Control group f (%)	*p*	Intervention group f (%)	Control group f (%)	*p*
**Age**						
< 20 years	2 (5)	2 (4)	0.911	0 (0)	0 (0)	0.712
20 – 35 years	36 (84)	39 (87)		31 (72)	35 (78)	
> 35 years	5 (12)	4 (9)		12 (28)	10 (22)	
**Education**						
Elementary- junior school	14 (33)	9 (20)	0.272	11 (26)	7 (16)	0.127
High school- Higher education	29 (67)	36 (80)		32 (74)	38 (84)	
**Income**						
< city minimum wage	24 (53)	15 (33)	0.056	24 (56)	15 (33)	0.368
> city minimum wage	19 (44)	30 (67)		19 (44)	30 (67)	
**Employment**						
Household/ non Job	34 (79)	35 (78)	0.582	1 (2)	2 (4)	0.140
Informal Sector	1 (2)	3 (7)		19 (44)	11 (24)	
Formal Sector	8 (19)	7 (16)		23 (54)	32 (71)	
**Health Insurances**						
Have Insurance	38 (88)	42 (93)	0.479	-	-	-
Don't Have Insurance	5 (12)	3 (7)				
**Parity**				-	-	-
0	12 (28)	20 (44)	0.260			
1 to 2	28 (65)	22 (49)				
> 2 Children	3 (7)	3 (7)				
**Information Exposure**						
Non digital	21 (49)	15(33)	0.207	7(16)	16 (36)	0.070
Digital	22 (51)	30 (67)		36(84)	29 (64)	

*Significant at *p* <0.05

**The effects of interventions on knowledge and attitudes**: Table 2 shows the effect of the intervention on increasing maternal (p=0.011; Δ=1.44) and husbands' knowledge (p=0.014; Δ=1.65) after 2.5 months of intervention. Likewise, the attitude towards post-partum care of the mother after the 3.5 month of intervention (p=0.023; Δ=4.01).

**Table 2:** The Effects of Interventions on knowledge and attitudes of mothers and husbands

Variable	Mothers				Husbands			

	Treatment Group	Control Group	Score difference	*p*	Treatment Group	Control Group	Score difference	*p*
	Mean (SD)	Mean (SD)			Mean (SD)	Mean (SD)		
Knowledge (score)								
Pre	22.0 (4.00)	22.2 (3.68)	-0.13	0,95	20.8 (4.63)	21.5 (4.04)	-0.68	0.46
Post I	23.9 (2.92)	22.4 (3.27)	1.44	**0.011** *	22.1 (3.45)	20.4 (3.34)	1.65	**0.014** *
Post II	26.7 (2.05)	25.4 (3.32)	1.23	0.120	24.8 (3.29)	24.7 (3.39)	0.03	0.88
Attitudes (score)								
Pre	85.5 (6.58)	87.8 (7.45)	-2.36	0.152	85.8 (6.15)	86.5 (6.91)	-0.72	0.74
Post I	89.5 (7.24)	88.9 (7.18)	0.62	0.85	86.3 (7.58)	87.2 (6.50)	-0.97	0.99
Post II	91.2 (5.81)	87.2 (9.64)	4.01	**0.023** *	90.0 (7.36)	87.6 (9.65)	2.40	0.26
Husbands' supports		-	-	-				
Post I	-	-	-	-	34.5 (1.79)	33.5 (3.38)	1.00	0.32
Post II	-	-	-	-	34.8 (1.74)	33.8 (3.43)	1.00	0.29

* Significant at *p* <0.05, pre=before intervention, post I=after 2.5 months intervention, post II=after 3.5 months intervention

**The effect of interventions on mother and husband's practices**: Table 3 shows the effect of the intervention on improving maternal practices regarding postpartum visits (p=0.001; RR=2.41; 95% CI=1.846–3.136), early initiation of breastfeeding (p=0.029, RR=1.68; 95% CI=1.120–2.523), consumption of iron tablet (p=0.005; RR=2.25; 95% CI=1.764–2.870), maintain personal hygiene (p=0.000; Δ=2.6), monitor danger signs of postpartum (p=0.001; Δ=1.2), enough consumption of nutritious food and drink (p=0.015; Δ=1.5) and husband's practice (p=0.000; RR=2.22; 95% CI=1.748–2.814).

**Table 3:** The effect of interventions on mother and husband's practices

Practice	Treatment Group	Control Group	p	RR	Confidence Interval	
					
	n=43	n=45			95%	
	Yes	Yes			Lower	Upper
	N (%)	N(%)			Limit	Limit
Mother's Practice						
Postpartum visits minimum 4 times	11 (26)	0 (-)	**0.001** *	2.41	1.846	3.136
Ask for early initiation of breastfeeding to medical workers	20 (47)	10 (22)	**0.029** *	1.68	1.120	2.523
Exclusive breastfeeding for 42 days	34 (79)	38 (84)	0.706	0.84	0.511	1.379
Consumption of iron tablet	7 (16)	0 (0)	**0.005** *	2.25	1.764	2.870
Consumption of vitamin A	38 (88)	32 (71)	0.081	1.95	0.900	4.243
Using post-partum contraception	21 (49)	27 (60)	0.403	0.80	0.520	1.218

**Mother's Practice**	**Median (Min-max)**	**Median (Min-max)**	**p**	**Score difference**		

Maintain personal hygiene and daily care	52.0 (48–57)	50.0 (39–55)	**0.000** *	2.6		
Monitoring for signs of postpartum danger	15.0 (10–15)	14.0 (7–15)	**0.001** *	1.2		
Consume enough nutritious food and drink	28.0 (22–33)	27.0 (22–32)	**0.015** *	1.5		

Husband's Practice	**Treatment Group**	**Control Group**	**P**	**RR**	**Confidence Interval**	
	**n=43**	**n=45**			**95%**	
	**Yes** **N (%)**	**Yes** **N (%)**			**Lower** **Limit**	**Upper** **Limit**

Husbands Accompanying mother for postpartum visits	6 (14)	0 (-)	**0.000** *	2.22	1.748	2.814

* Significant in *p*<0.05

## Discussion

The m-Health interactive message intervention increased the knowledge of mothers and husbands after 2.5 months of intervention. The intervention was carried out every day, for 5 hours per day continuously until the 14th day, accompanied by interactive assistance in the form of consultation, sharing, discussion, and question and answer, making it easier for mothers to remember, understand, and recognize the information that has been given. Thus, the intervention increased the mothers' and husbands' knowledge on postpartum care. In addition, promotional media in research in the form of text messages, images, and videos, make it easier for subjects to understand, remember, and imitate the information obtained.

Increased knowledge as a result of the intervention is a factor that influences the change of a person's attitude toward the positive attitude. In addition, changes in positive attitudes are also caused by the existence of communication, interaction, social networks, and the impact of the use of social media (15). In this study, it was seen that there was an increase in the mothers' attitude after 3.5 months of intervention.

Previous studies on mobile phones to increase knowledge in maternal and child health care have been reported in Brazil (16) and Bangladesh (17). The difference with this study is the increase in maternal knowledge after 2.5 months of intervention, while the research in Bangladesh took six months to had a good pattern of acceptance (receiving and listening to messages sent). Meanwhile, the husbands' involvement in maternal and child health also impacts the husband's knowledge about the danger signs of pregnancy and postpartum and about the preparation for childbirth (18). The difference with this research was in the use of promotional media, in which this study used the online method.

Interventions give an increase in postpartum visits. Mothers who received the intervention had a 2.41 times higher chance of having a postpartum visit than those who did not receive the intervention. This is in accordance with previous research in Nepal, where health education for husband and wife increased antenatal care visits and postpartum visits (19). A similar study was carried out in Brazil, which used text messages to increase antenatal care visits. The similarity with this research was the use of text message media (16).

The intervention increased the chances of mothers asking for early breastfeeding initiation to medical workers by 1.68 times higher than those who did not receive the intervention. This result was similar to a study in Nigeria on 390 mothers, where the use of text and voice messages had a 2.6 times chance to initiate timely initiation of breastfeeding and increase exclusive breastfeeding up to 6 months (20). In addition, a meta-analysis study on cell phone use showed an increase in early initiation of breastfeeding within 1 hour after birth (21).

The chance of consuming iron tablets was 2.64 times higher in mothers who were given the intervention than the mothers who did not receive the intervention. This result was similar to a study in Kenya regarding the use of text messages for six months, which increased the consumption of iron tablets (22). However, in this study, the intervention for 3.5 months had shown a positive effect on iron tablets' consumption.

The intervention had improved the mothers' practice in monitoring postpartum danger signs of bleeding, lochia fluid, breast conditions, and health problems such as fever and prolonged sadness. These results were supported by previous research. For example, educational programs for rural pregnant women in Egypt increased 63% of mothers' knowledge and practices in monitoring pregnancy danger signs (23). Meanwhile, in Tanzania, men's home base saving skills intervention increased knowledge of at least mentioning three dangerous symptoms of postpartum such as heavy bleeding, foul-smelling vaginal discharge, abdominal pain, weakness, and breathing difficulty (18).

Our research also showed the improvement in the mother's practice of maintaining daily hygiene, such as: regular bathing, washing hands with running water and soap when defecating and urinating, before handling the baby and before eating, maintaining the cleanliness of the vulva and perineum, and changing sanitary napkins regularly. A study in Bangladesh showed that 16 days of counseling increased the availability of soap hand washing stations in the baby's sleeping area and increased the practice of mothers washing their hands at the recommended times (24). While the findings of a mixed study in Nigeria found that good hand hygiene practices were observed in mothers, caregivers other than mothers, and health workers accounted for only 1% of all hand hygiene practices. Good hand hygiene practices are quite low, 6% in health care facilities and 7% in households (25).

This study proved an increase in mothers' practice in consuming nutritious food and adequate drinks after the intervention. The same thing was shown in a study in America that increased the vegetable intake during the postpartum period after receiving nutrition education through face-to-face and pamphlets (26).

This study did not improve the mother's practice of giving exclusive breastfeeding for 42 days. Many factors influenced exclusive breastfeeding for 42 days in addition to the interventions that had been given. Despite having good knowledge about exclusive breastfeeding, new mothers were not necessarily going to put it into practice. Medical reasons such as sore nipples and mastitis made them stop breastfeeding (27). The rejection by the mothers, time insufficiency, husband's disagreement, the perception that exclusive breastfeeding was not required was the inhibiting factors for exclusive breastfeeding (28). In this study, most of the subjects were 20–35 years of age, and their parity was between 0–2 children. This might be the reason for the failure of the intervention in this study to increase exclusive breastfeeding. The findings in Zimbabwe showed that the inability of exclusive breastfeeding was due to parity (1–2 children) and young mothers (< 25 years old) (29), besides cultural factors, environment, family support, nutrition, and psychology associated with exclusive breastfeeding (30). In this study, several obstacles that are thought to have an impact on the practice of exclusive breastfeedings, such as social pressure from families and the environment to provide complementary foods (31), maternal psychological pressure during the COVID-19 pandemic in the form of stress and anxiety (32), and concerns about breastfeeding safety during the postpartum period for infected or suspected COVID-19 mothers (33).

The intervention in this study also failed to improve the practice of using postpartum contraception after delivery. The 2014 National Socio-Economic Survey results showed that the users of contraceptives in Indonesia were working mothers, aged 30–34, living in urban areas (34). This condition was in accordance with the characteristics of the subjects, wherein the treatment group, 80% were housewives/nonworking mothers who lived in suburban areas. Meanwhile, in Ethiopia, factors that influence the use of postnatal contraceptives other than knowledge are the return of menstruation, husband's consent, starting sex, and the 6–12 month postpartum period (35).

Increased knowledge of the husbands did not improve the husband's positive attitude on postpartum care. According to Albarracin, changes in a person's positive attitude are related to the cultural and emotional context (15). The observations on the mentoring process during the intervention showed that most husbands were not active in the discussion process on WhatsApp groups compared to mothers. In this case, it was unknown whether the sent message had been read and studied yet. There is still a perception that maternal and child health is a woman's business because women are better at handling pregnancy, childbirth, and the puerperium. In addition, husbands felt embarrassed and uncomfortable to discussing issues related to femininity. The same pattern occurred in a study in Nigeria regarding the low involvement of husbands during pregnancy, childbirth, and postpartum, which was caused by strong social norms and gender roles (36). The picture of the husband's inactivity during the study was seen in the husband's support which did not increase after 2.5 months and 3.5 months of intervention. The results of this study were different from previous studies related to the husband's involvement in the use of cell phones to have a better impact on the use of contraception (37), nutritional support (38), and monitoring the danger signs of pregnancy (18).

In this study, the intervention increased the practice of husbands accompanying mothers during postpartum visits. In this case, the increase in knowledge obtained from the intervention did not foster a positive attitude of the husband on postpartum care but was directly realized in the form of practice. This is evidenced by the number of husbands who practice in the treatment group (14.0% vs. 0.0%). These results are similar to previous studies in Indonesia (39), India (40), and Nepal (19).

In conclusion, this study increased most of the postpartum care behaviors of mothers and husbands. The increases were in mothers' knowledge, attitudes, and practices related to postpartum visits, early breastfeeding initiation, iron tablets consumption, personal hygiene, nutritious food consumption, adequate drinks, and danger signs of postpartum monitoring. Therefore, m-health interactive messages are recommended to improve mothers and husbands' behavior in postpartum care, especially during the COVID-19 pandemic.

Not all of the subjects were actively involved in WhatsApp groups (intervention was given in the WhatsApp group). Some of the subjects did not respond to the sent messages. In this study, there was no mechanism to control whether the messages sent were learned, practiced, or simply opened. Some husbands were embarrassed to ask questions and believed that maternal and child care were the women's issues.
